# Supplementary material for: Analysis of SI-Related BoGAPDH Family Genes and Response of BoGAPC to SI Signal in Brassica oleracea L
Source: Genes (Basel). 2021 Oct 28;12(11):1719. doi: 10.3390/genes12111719 (PMC8618600; doi:10.3390/genes12111719)
Supplement: Supplementary file 1 [file genes-12-01719-s001.zip › genes-1398301-supplementary.pdf]

Table S1. Primers used in gene cloning.

Primer name	Primer sequence (5→3)	Functions
BoGAPC-AD	F: GTACCAGATTACGCTCATATGGCTGACAACAAGAAGATTAAG<br>R: ATGCCCACCCGGGTGGAATTCTTAGGCTTTGGACATGTGGATG	Yeast two-hybrid primers
SRKj-AD	F: GTACCAGATTACGCTCATATGTCAGATCGTGAGGTTTTCAATTC<br>R: ATGCCCACCCGGGTGGAATTCGCCTTGTTATCGTTCCATGACTA	Yeast two-hybrid primers
BoGAPC-PAN	F: AAGTCCGGAGCTAGCTCTAGAGCTGACAACAAGAAGATTAAG<br>R: GCCCTTGCTCACCATGGATCCGGCTTTGGACATGTGGATG	Subcellular location
BoGAPC-PGEX	F: GATCTGGTTCCGCGTGGATCCGCTGACAACAAGAAGATTAAG<br>R: GATGCGGCCGCTCGAGTCGACGGCTTTGGACATGTGGATG	Prokaryotic expression
BoGAPC-GUS	F: CAAGCTTGGCTGCAGGTCGACAACCATGCATAACACAAATAACC<br>R: GGTGGACTCCTCTTAGAATTCGGCGATGAGATCGAGAGAGA	Promoter activity analysis

Table S2. Primers used in RT-PCR.

Target gene symbol	RT-PCR target- length	accession number	Primer sequence (5→3)
BoActin	124	XM_013780726.1	F: CCAGAGGTCTTGTTCCAGCCATC<br>R:GTTCCACCACTGAGCACAATGTTA
Bo5g021670.1	157	XM_013728196.1	F: AAAACTAACGGGAATGGCTTTC<br>R: TTCTGTGTACCCCATAATTCCC
Bo8g065470.1	94	XM_013749462.1	F: CGCTGTTAACGATCCTTTCATC<br>R: CTTAAGCTCATTGTGCTTCCAC
Bo5g148380.1	92	XM_013782360.1	F: GTCCACTCTATCACTGCTACTC<br>R: GGAATGATGTTGAAGGAAGCAG
Bo8g107420.1	132	XM_013744693.1	F: CTATATTCTTCACGCTCGACCT<br>R: TCCTAATCTTCTTGTCAGCCATT
Bo6g080970.1	124	XM_013733000.1	F: CTGCTACTCAGAAGACTGTTGA<br>R: TCTGGCAGAACTTTACCTACAG
Bo02686s010.1	96	XM_013759962.1	F: CAGCCGATCTCACTGCATAC<br>R: GCACTTCTGTCTTTGCAACC
Bo2g095350.1	124	XM_013762641.1	F: CTGCTACTCAGAAGACTGTTGA<br>R: TCTGGCAGAACTTTACCTACAG
Bo5g016460.1	89	XM_013731565.1	F: ACTTACGTTTGTGGTGTCAATG<br>R: GGAGCGAGACAGTTAGTAGTAC
Bo8g024290.1	118	XM_013749055.1	F: AACCTATGTTGTGGGAGTCAAT<br>R: CTCTTCATCCAAAACTTTCGCA
Bo5g017500.1	81	XM_013731507.1	F: TTGAGTCTACTGGTGTCTTCAC<br>R: CAGAGATGACAACTTTCTTCGC

Bo8g107880.1	89	XM_013745604.1	F: ACTTACGTTTGTGGTGTCAATG<br>R: GGAGCGAGACAGTTAGTAGTAC
Bo00285s370.1	124	XM_013757977.1	F: TGCTTACAATCCTGATGAACCT<br>R: GTGAGTTGTTGTCATTGTACCC
Bo3g057250.1	97	XM_013766979.1	F: CCTCGGATCTCACTCAAACC<br>R: CCGATTCTTCCGAATCCGTT
Bo1g152180.1	132	XM_013773250.1	F: CGATACACTCACCATGGCTAAC<br>R: ATGAAGGGATCGTTGACAGC

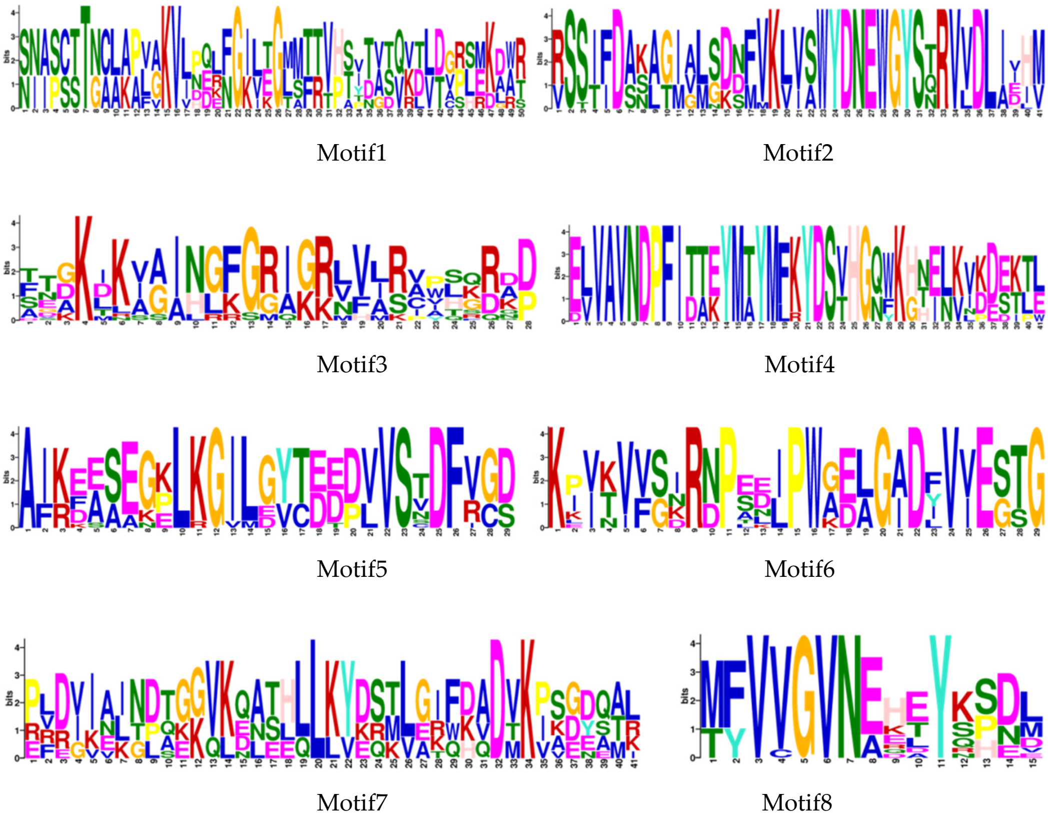

Figure S1. The sequence logos of *GAPDH* family genes in *B. oleracea.*

Motif	*E*-value	Sites	Width	Motif	*E*-value	Sites	Width
1	5.6×10^{-566}	22	50	6	9.3×10^{-170}	13	29
2	2.3×10^{-321}	13	41	7	1.6×10^{-81}	9	41
3	1.7×10^{-212}	22	28	8	$3.8e\times10^{80}$	13	15
4	7.4×10^{-211}	9	41	9	1.1×10^{-63}	4	50
5	5.4×10^{-172}	13	29	10	2.8×10^{-55}	8	29

Table S3. Conservative domains and amino acids of *GAPDH* family genes in *B. oleracea.*

Table S4. KaKa analysis.

Gene pairs	Ka	Ks	Ka/Ks	*P*-Value(Fisher)
Bo5g021670.1&Bo6g080970.1	0.0564364	0.758076	0.0744468	5.26×10^{-75}
Bo5g021670.1&Bo2g095350.1	0.0620217	0.716155	0.0866037	3.05×10^{-68}
Bo5g148380.1&Bo1g152180.1	0.00634094	0.243379	0.0260537	4.64×10^{-28}
Bo5g148380.1&Bo3g057250.1	0.00785431	0.251522	0.0312271	5.23×10^{-28}
Bo5g148380.1&Bo5g017500.1	0.0161833	0.406074	0.039853	4.12×10^{-40}
Bo5g148380.1&Bo8g065470.1	0.0186877	0.449185	0.0416036	2.32×10^{-43}
Bo6g080970.1&Bo2g095350.1	0.0340282	0.257343	0.132229	5.12×10^{-24}
Bo8g065470.1&Bo5g017500.1	0.00418655	0.214589	0.0195096	4.97×10^{-26}
Bo8g065470.1&Bo3g057250.1	0.0153723	0.500133	0.0307364	7.39×10^{-49}
Bo8g065470.1&Bo1g152180.1	0.986509	1.04741	0.941858	0.209309
Bo5g016460.1&Bo8g107880.1	0.00813763	0.296307	0.0274635	2.04×10^{-40}
Bo5g016460.1&Bo00285s370.1	0.0424474	0.823045	0.0515737	1.76×10^{-80}
Bo5g017500.1&Bo3g057250.1	0.0143038	0.512163	0.0279283	6.08×10^{-49}
Bo5g017500.1&Bo1g152180.1	0.968431	1.11235	0.870618	0.00142489
Bo8g107880.1&Bo00285s370.1	0.045572	0.831179	0.0548282	1.43×10^{-80}
Bo3g057250.1&Bo1g152180.1	0.978915	1.06386	0.920155	0.0530145

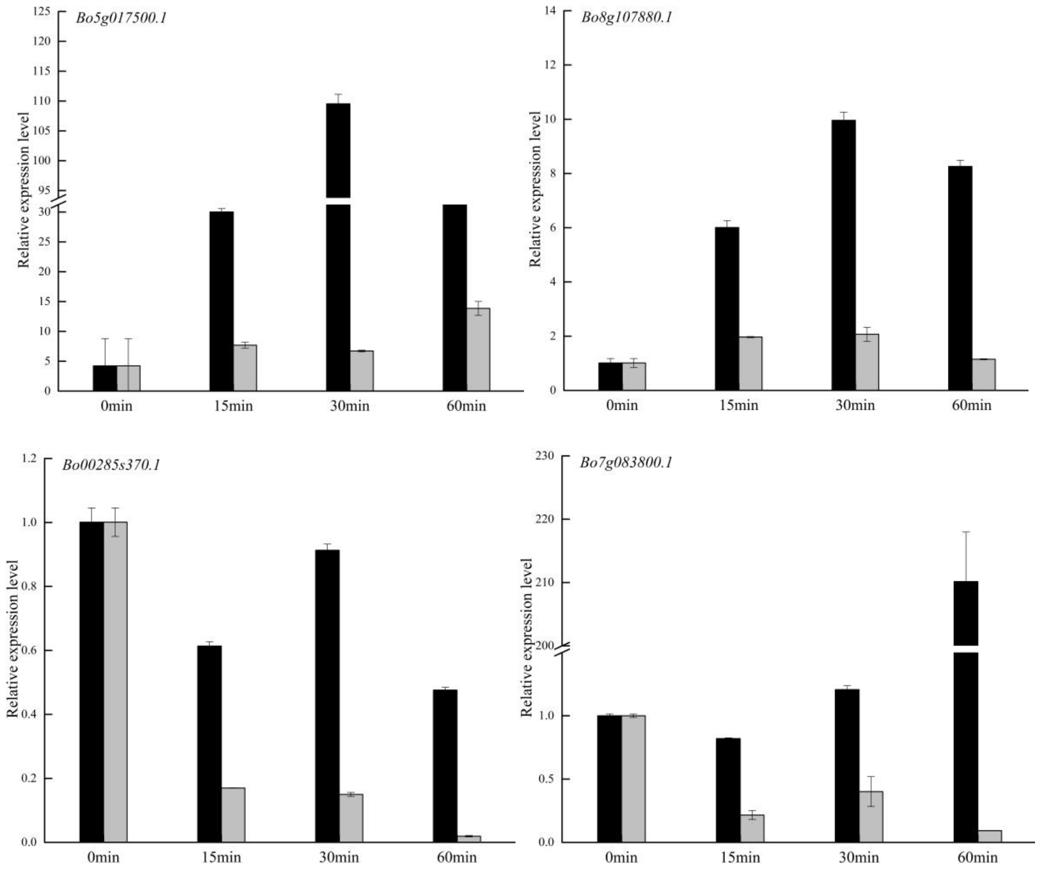

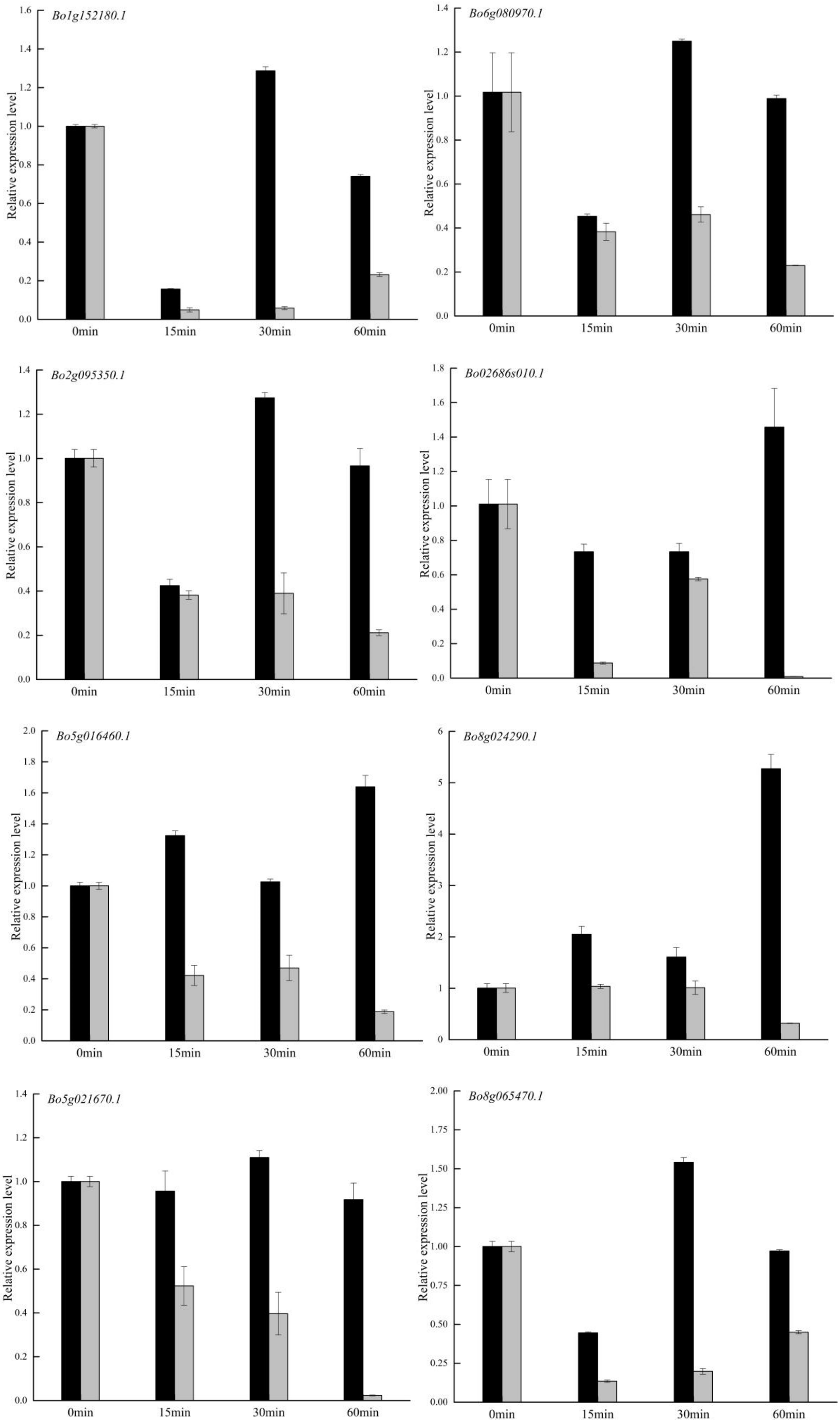
Bo1g152180.1
Bo6g080970.1
Bo2g095350.1
Bo02686s010.1
Bo5g016460.1
Bo8g024290.1
Bo5g021670.1
Bo8g065470.1
Relative expression level
0min
15min
30min
60min

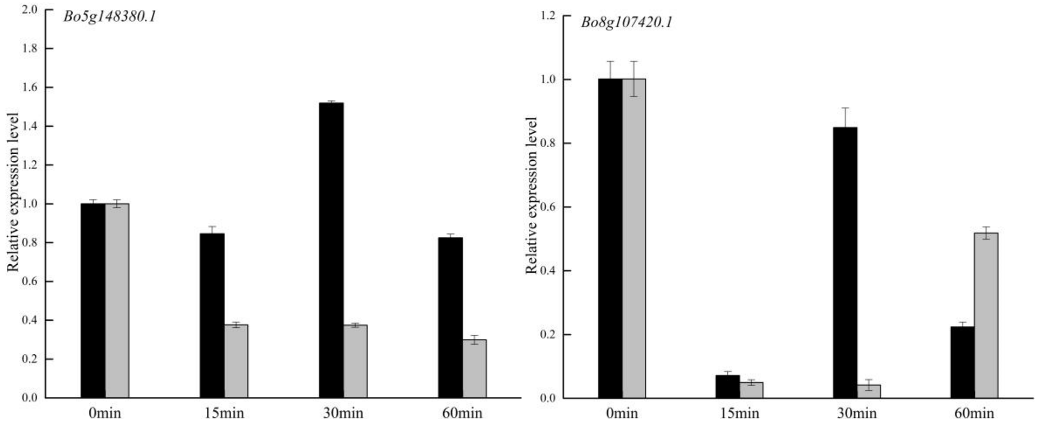

Figure S2. *BoGAPDH* expression analysis after pollination. The black column indicates compatible pollination, and the gray column indicates incompatible pollination. The pollination period is 0 min, 15 min, 30 min, 60 min.

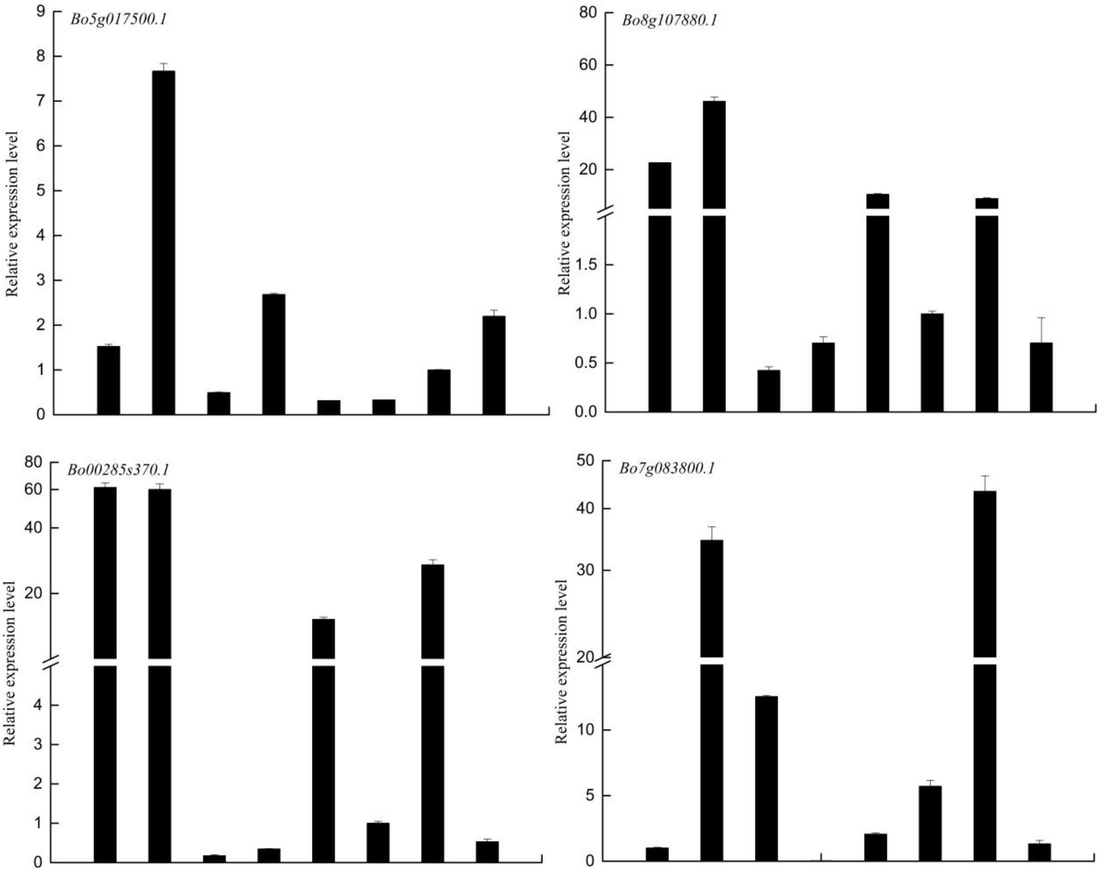

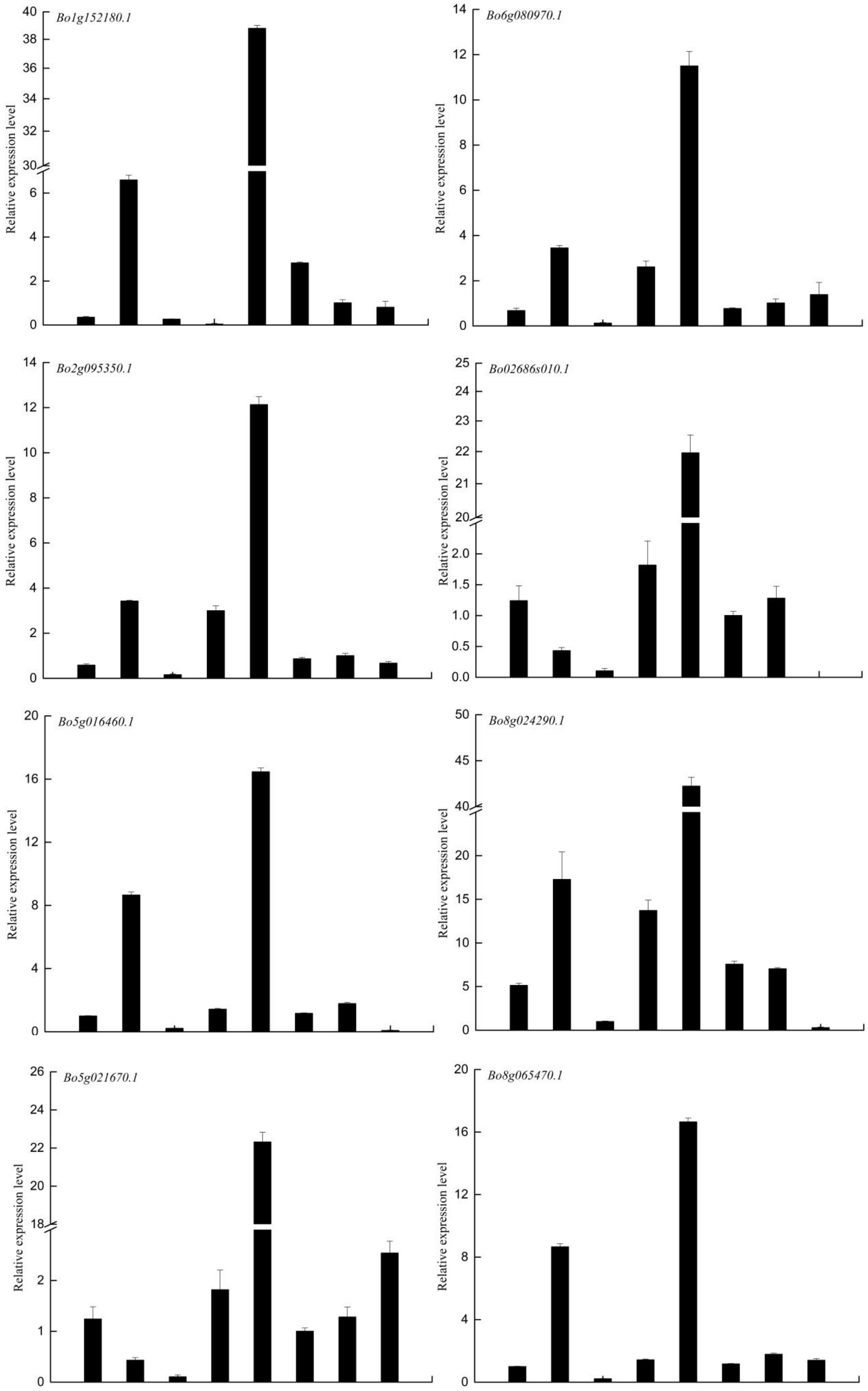
Bo1g152180.1
Bo6g080970.1
Bo2g095350.1
Bo02686s010.1
Bo5g016460.1
Bo8g024290.1
Bo5g021670.1
Bo8g065470.1
Relative expression level
40
38
36
34
32
30
6
4
2
0
14
12
10
8
25
24
23
22
21
20
2.0
1.5
1.0
0.5
0.0
16
50
45
15
5
26
18

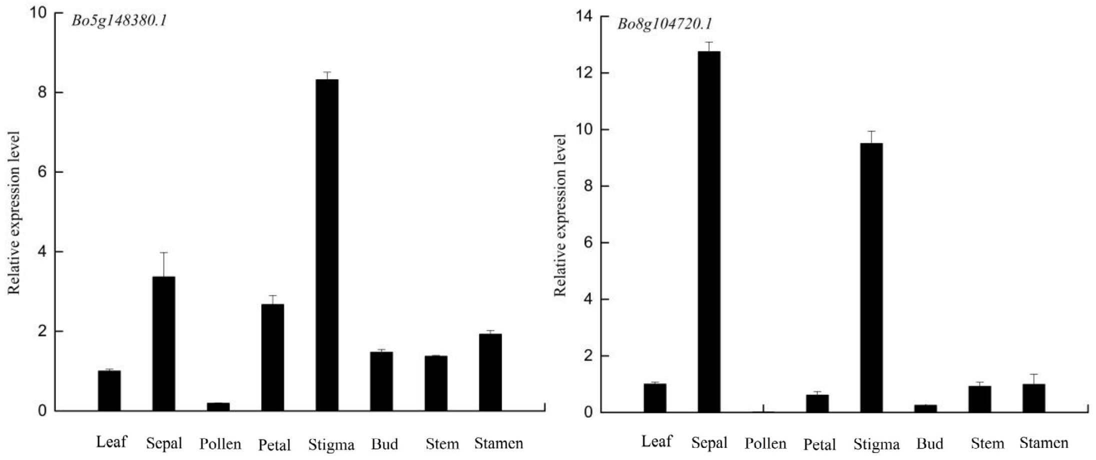

Figure S3. Tissue Expression Analysis of *BoGAPDH. B. oleracea* tissue includes leaves, sepal, pollen, petals, stigma, bud, stem and stamen.

```
  1 ATGGCTGACAACAAGAAGATTAAGATCGGAATCAACGGTTTCGGAAGAATCGGTCGTTTGGTGGCGAGAGTTATCCTTCAGAGGAACGAT
    M  A  D  N  K  K  I  K  I  G  I  N  G  F  G  R  I  G  R  L  V  A  R  V  I  L  Q  R  N  D
 91 GTTGAGCTCGTCGCTGTTAACGATCCTTTCATCACCACCGAGTACATGACGTACATGTTTAAGTACGACAGTGTTCACGGGCAGTGGAAG
    V  E  L  V  A  V  N  D  P  F  I  T  T  E  Y  M  T  Y  M  F  K  Y  D  S  V  H  G  Q  W  K
181 CACAATGAGCTTAAGATAAAGGATGAGAAAACACTTCTCTTTGGCGAGAAGCCAGTCACCGTGTTCGGCATCAGGAACCCTGAGGAGATC
    H  N  E  L  K  I  K  D  E  K  T  L  L  F  G  E  K  P  V  T  V  F  G  I  R  N  P  E  E  I
271 CCATGGGGTGAGGCCGGAGCTGACTTTGTTGTTGAGTCTACTGGTGTTTTCACTGACAAGGACAAGGCCGCTGCTCACTTGAAGGGTGGT
   P  W  G  E  A  G  A  D  F  V  V  E  S  T  G  V  F  T  D  K  D  K  A  A  A  H  L  K  G  G
361 GCCAAGAAAGTTGTCATCTCTGCTCCAAGCAAAGATGCTCCCATGTTTGTTGTTGGTGTCAATGAGCATGAATACAAGTCTGATCTTAAC
    A  K  K  V  V  I  S  A  P  S  K  D  A  P  M  F  V  V  G  V  N  E  H  E  Y  K  S  D  L  N
451 ATTGTTTCCAACGCTAGTTGCACCACTAACTGCCTTGCTCCACTTGCCAAGGTTATTAACGACAGGTTTGGAATTGTTGAGGGACTCATG
    I  V  S  N  A  S  C  T  T  N  C  L  A  P  L  A  K  V  I  N  D  R  F  G  I  V  E  G  L  M
541 ACTACCGTCCACTCTATCACTGCTACTCAAAAGACTGTTGATGGTCCATCAATGAAGGACTGGAGAGGTGGAAGAGCCGCTTCCTTCAAC
    T  T  V  H  S  I  T  A  T  Q  K  T  V  D  G  P  S  M  K  D  W  R  G  G  R  A  A  S  F  N
631 ATCATTCCCAGCAGCACCGGAGCTGCCAAGGCTGTCGGAAAGGTGCTTCCACAGCTCAACGGAAAGCTGACCGGAATGTCCTTCCGCGTT
    I  I  P  S  S  T  G  A  A  K  A  V  G  K  V  L  P  Q  L  N  G  K  L  T  G  M  S  F  R  V
721 CCCACCGTTGATGTTTCAGTTGTTGACCTCACGGTTAGACTCGAGAAAGCTGCGACCTACGATGAAATCAAGAAGGCTATCAAGGAGGAA
    P  T  V  D  V  S  V  V  D  L  T  V  R  L  E  K  A  A  T  Y  D  E  I  K  K  A  I  K  E  E
811 TCTGAAGGCAAGCTAAAGGGAATCCTTGGTTACACCGAGGATGATGTTGTCTCAACTGACTTCGTTGGTGACAACAGGTCGAGCATTTTT
    S  E  G  K  L  K  G  I  L  G  Y  T  E  D  D  V  V  S  T  D  F  V  G  D  N  R  S  S  I  F
901 GACGCAAAGGCTGGAATTGCGTTGAGTGACAACTTCGTGAAACTGGTGTCGTGGTATGACAATGAATGGGGTTACAGTACCCGTGTGGTC
    D  A  K  A  G  I  A  L  S  D  N  F  V  K  L  V  S  W  Y  D  N  E  W  G  Y  S  T  R  V  V
991 GACTTGATCATCCACATGTCCAAAGCCTAA
    D  L  I  I  H  M  S  K  A  *
```

Figure S4. BoGAPC cDNA and its amino acid sequence. The first row represents the sequence of the bases, and the second row represents the amino acids corresponding to the bases, the underlined part is the conservative domain of Gp_dh_N.

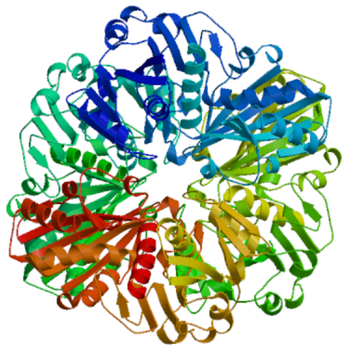

Figure S5. Prediction of three-dimensional structure of BoGAPC protein.

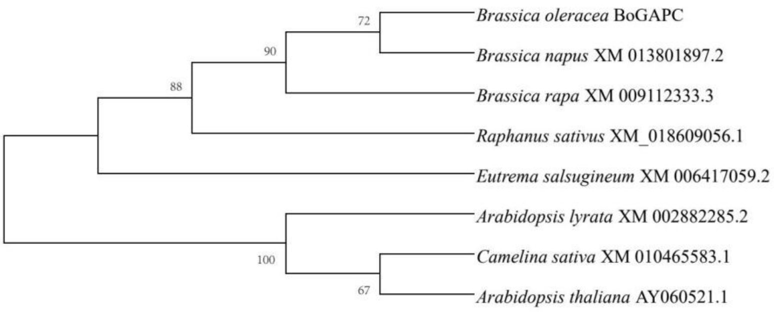

Figure S6. Phylogenetic tree of BoGAPC and other species GAPC protein.

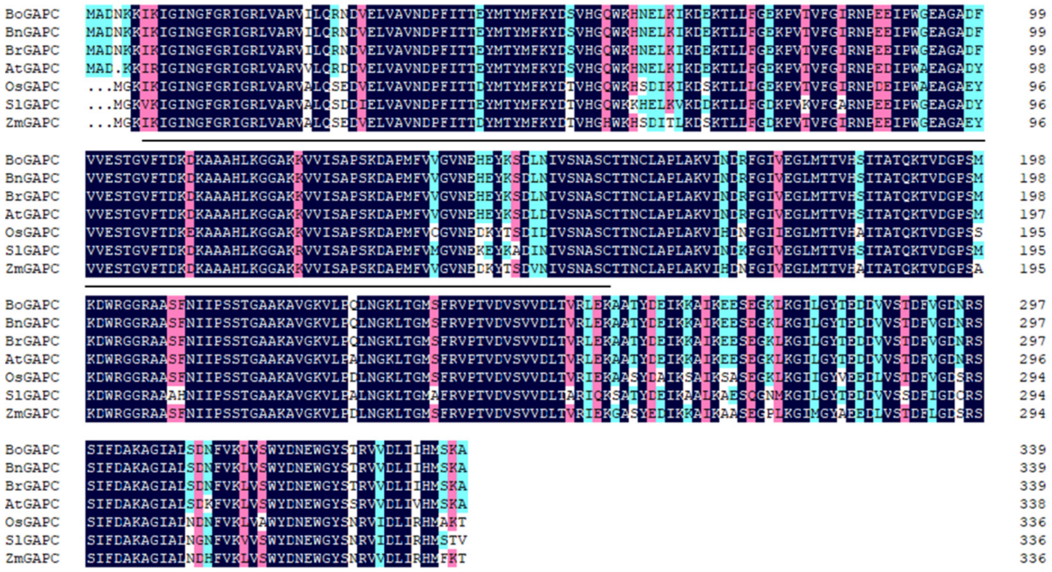

Figure S7. Alignment of BoGAPC of *B. oleracea* with homologous proteins of other species. Black: amino acid identity 100%; Pink: amino acid identity 75%; Blue: amino acid identity 50%, the underlined part is the conservative domain of Gp_dh_N.

Table S5. Cis-elements in the upstream regulation region of BoGAPC.

Associated putative function	Cis-elements in the promoter region
Abscisic acid response	ABRE、MYCATRD22、DPBFCOREDCD3、MYC CONSENSUSAT
Gibberellin-response	WEKY710S
Ethylene-response	ERELEE4
pollen-specific cis-acting element	POLLENLELAT52、MYBPLANT、GTGANGTG 10
Stress and defense related response elements	WBOXATNPR1、WBOXNTERF3
Carbon metabolism related response elements	POLASIG1、DOFCOREZM、WBOXHVISO1、C ACTFTPPCA1
Light response	EBOXBNNAPA、IBOXCORE、DPBFCOREDCD C3
Cytokinin response	ARR1AT
Pigment related response element	REALPHALGLHCB21

S1. Materials and Methods

S1.1. RNA extraction and reverse transcription reaction

The kit we used for RNA extraction was provided by Sangon Biotech (Shanghai) Co., Ltd. under model number NO. B518631, we strictly follow the All-in-One First-Strand cDNA Synthesis SuperMix for qPCR (One-Step gDNA Removal) (Model No. AE341-02) provided by TransGen Biotech for The details of the reverse transcription reaction, the concentration of RNA used, the volume and the temperature and time of the reaction can be found in the kit instructions and are briefly summarized as follows.

RNA was extracted and stored at -80 °C according to the instructions of the Plant Total RNA Extraction Kit (Shanghai Bioengineering Co., Ltd.).

1. Add 600 μL of Buffer Rlysis-P to a 1.5 ml RNase-free centrifuge tube and set aside.
2. Take 25-50 mg of plant tissue and grind it into powder with liquid nitrogen, add it to the above 1.5 mL centrifuge tube, and immediately shake and mix.
3. Lysis the sample completely in a 65°C water bath for 5 min.
4. Add 60 μL of Buffer PCA to the lysed sample and mix well. -Leave at 20°C for 3 min.
5. Centrifuge at 12,000 rpm for 5 min at room temperature at 4°C and remove supernatant.
6. Add an equal volume of phenol: chloroform (25:24, pH 4.5) to the supernatant and mix well. 12,000 rpm 4°C centrifuge for 5 min and remove the supernatant.
7. Add an equal volume of chloroform to the supernatant and mix well. 12,000 rpm 4°C centrifuge for 5 min and remove the supernatant.
8. Add 1/3 volume of anhydrous ethanol, mix well, leave at room temperature for 3 min, centrifuge at 12,000 rpm for 5 min at 4°C, and carefully pour off the supernatant.
9. Wash the precipitate with 700 μL of 75% ethanol (prepare with DEPC-treated ddH2O), centrifuge at 12,000 rpm for 3 min at 4°C, and carefully pour off the supernatant.
10. Repeat step 9 once. 11.
11. Invert the tube to room temperature for 10 min to allow as much ethanol as possible to evaporate. Add 50 μL of DEPC-treated ddH2O to dissolve the precipitate for immediate use or long-term storage at -80°C.

Using the above-mentioned RNA as a template, the first strand of cDNA was synthesized and stored at -80 °C. The specific steps of the kit (TransGen, Beijing) are as follows.

1. Add the following kit samples according to the instructions in the following system.

Reagent name	Usage
Total RNA	1 μg

Random Primer	1 μL
2×ES Reaction Mix	10 μL
EasyScript RT/RI Enzyme Mix	1 μL
gDNA Remover	1 μL
Rnase-free Water	To 20 μL

2. Mix the solutions of the above reactions, and incubate them at 42 ℃ for 15 min if the subsequent experiment is with cDNA from qPCR experiments; incubate the fragments of cDNA used to amplify genes at 42 ℃ for 30 min.
3. After the above procedure is completed, the inversion sample tube is heated at 85 ℃ for 5 s. The purpose of this operation is to inactivate EasyScript RT/RI and gDNA Remover in the mixed system.
4. The cDNA after the reaction is completed will be tested for the quality of the template with B. oleracea Action.

S1.2. RT-PCR methods

We performed RT-PCR experiments using the B Green® Premix Ex Taq™ II (Tli RNaseH Plus) kit provided by Takara Biomedical Technology (Beijing) Co., Ltd. The volumes required for the reactions are shown below.

Reagents	Usage amount	Final concentration
TB Green Premix Ex Taq II（Tli RNaseH Plus）（2X）	12.5 μL	1X
PCR Forward Primer（10 μM）	1 μL	0.4 μM
PCR Reverse Primer（10 μM）	1 μL	0.4 μM
DNA Template（80ng/μL)	2 μL	
H2O	8.5 μL	
Total	25 μL	

The conditions required for the reaction are shown below.

Step 1：95℃ 30 s

Step 2：PCR Reaction

GOTO：39（40 Cycles）

95℃ 5 s

60℃ 30 s

Step 3：Melt Curve

Details of concentration of magnesium ions, dNTPs, DNA polymerase type, and concentration can be found in the kit.
